# Zebra leaf 15, a receptor-like protein kinase involved in moderate low temperature signaling pathway in rice

**DOI:** 10.1186/s12284-019-0339-1

**Published:** 2019-11-15

**Authors:** Ping Feng, Junqiong Shi, Ting Zhang, Yuqin Zhong, Lisha Zhang, Guoling Yu, Tianquan Zhang, Xiaoyan Zhu, Yadi Xing, Wuzhong Yin, Xianchun Sang, Yinghua Ling, Changwei Zhang, Zhenglin Yang, Guanghua He, Nan Wang

**Affiliations:** Rice Research Institute, Key Laboratory of Application and Safety Control of Genetically Modified Crops, Academy of Agricultural Sciences, Southwest University, Chongqing, 400715 China

**Keywords:** Zebra leaf, Receptor-like protein kinase, Moderate low temperature, Rice

## Abstract

**Background:**

Zebra leaf mutants are an important resource for studying leaf colour in rice. In most such mutants, the zebra leaf phenotype results from defective chloroplast biogenesis. The molecular mechanism by which zebra leaves develop remains unclear, so additional zebra-leaf mutants need to be identified.

**Results:**

We isolated a novel rice zebra-leaf mutant, named *zebra leaf 15* (*z15*), which showed transversely striped leaves with yellow-green or white-green sectors, in which chloroplast structure was disturbed. Transmission electron microscopy revealed that the structure of various organelles was impaired in yellow/white sectors. *Z15*, a single-copy gene in the rice genome, encodes a receptor-like protein kinase. Subcellular localization analysis indicates that Z15 and z15 are localized on the plasma membrane. The expression of *Z15* is induced by moderate low temperature (18 °C). The mutation of *Z15* influenced the expression of two downstream genes, *OsWRKY71* and *OsMYB4*, that were responsive to moderate low temperature. The results show that *Z15* plays a crucial role in the early stages of the response to moderate low temperature in rice.

**Conclusions:**

We identified a novel zebra-leaf mutant (*z15*) that impaired chloroplast structure in rice, *LOC_Os05g12680*, encoding a receptor-like protein kinase. Further study indiceted that *Z15* plays a crucial role in the early stages of the response to moderate low temperature in rice.

## Background

The “zebra leaf” trait of rice manifests as regular intermittent chlorosis of the leaves and sheaths. The entire leaf shows transverse green/yellow-striped segments, which are similar to zebra striping patterns. Under certain conditions, the zebra pattern may fade gradually and the leaves assume a normal appearance. Temperature is one important environmental factor that affects the zebra leaf trait in rice. The temperature-sensitive mutant *tsc1* shows a white, light-yellow, and normal green leaf when grown at 23.1 °C, 26.1 °C, and 30.1 °C, respectively (Dong et al. 2001). The temperature-conditioned mutants *v3* and *st1* produce bleached leaves at constant 20 °C or 30 °C, but leaves that are almost green develop under diurnal 30 °C/20 °C conditions (Yoo et al. 2009). These studies found variations in leaf colour at different temperatures. However, no-one has yet conducted a systematic investigation of the temperature-sensing mechanism of zebra leaf mutants.

Low temperature is a major environmental factor that limits plant growth, which is classifiable into chilling (0–15 °C) and freezing (< 0 °C) temperatures based on plant tolerance (Zhou et al. 2011). The most frequently applied low-temperature treatment is 4 °C, to which many genes are reportedly responsive; examples are *OPEN STOMATA 1* (*OST1*), *BRASSINAZOLE RESISTANT 1* (*BZR1*), and *COLD RESPONSIVE PROTEIN KINASE 1* (*CRPK1*) (Ding et al. 2015; Li et al. 2017; Liu et al. 2017). Responses to moderate temperature (15–28 °C) are rarely studied. Many plant species have evolved a series of mechanisms that minimize the negative effects of cold stress. These mechanisms are divided mainly into CBF-dependent and CBF-independent pathways. The CBF transcriptional regulatory cascade plays important roles in plant response to cold. *CRPK1*, a cold-activated plasma membrane protein, is involved in the CBF-dependent pathway. *CRPK1* phosphorylates 14–3-3 proteins, then the phosphorylated 14–3-3 proteins are transported from the cytosol to the nucleus, and ultimately through the CBF pathway through which the cold-response signal is transmitted (Liu et al. 2017). *CHILLING TOLERANCE DIVERGENCE 1* (*COLD1*) is an additional CBF-dependent pathway regulator that encodes a regulator of G-protein signalling. Interaction of COLD1 and its protein partner RGA1(rice G-protein α subnit 1) triggers a calcium influx that leads to the activation of crucial CCAAT motif-binding factor (CBF) transcription factors for chilling tolerance in rice (Ma et al. 2015). In addition to the CBF-dependent pathway, many genes involved in low-temperature regulation belong to CBF-independent pathways. The transcription factors *MYB4* and *MYB3R2* in rice can regulate the response to cold stress positively, but the mechanism is different from that of the CBF pathway (Dai et al. 2007; Vannini et al. 2004). *BZR1* may act as a cross-regulator to regulate the cold response and other signalling pathways. In the non-CBF pathways, *BZR1* binds directly to the promoter of the CBF-independent cold-regulated genes, such as *WRKY6*, *PYL6*, *SOC1*, *SAG21*, and *JMT*, which regulate the cold response (Li et al. 2017). Information on CBF-independent pathways remains incomplete. Therefore, identification of additional genes involved in CBF-independent pathways is needed to provide a deeper understanding of the mechanism of the response to low temperature.

The receptor-like protein kinases (RLKs) gene family is one of the largest gene families in plants. It plays an important role in the regulation of plant growth and development, signalling networks, and disease resistance (Ye et al. 2017). Plant RLKs are involved in the response to cold stress and mediate signal transduction. A calcium-regulated RLK, *CRLK1*, modulates acclimation to cold by means of a MAP kinase cascade in plants and promotes the expression of cold-stress response genes, and ultimately plant adaptation to low temperature is regulated (Yang et al. 2010a; Yang et al. 2010b). Proline extension receptors (PERKs) are a class of plant RLKs with an extracellular domain rich in proline. Limited research on the PERK family has been conducted. *PERK1* encodes a putative receptor protein kinase in *Brassica napus* L. and shows a similar structure to wall-associated kinases (WAKs). *PERK1* plays a role in mediation of early events in the plant defence response to mechanical injury, perhaps by sensing changes in the cell wall via its extension-like extracellular domain, and a wound-response signalling cascade is triggered through its catalytic domain (Silva and Goring 2002). *AtPERK4*, which encodes a receptor-like kinase, plays a role in the early stages of abscisic acid (ABA) signalling to inhibit primary root cell elongation by perturbation of Ca^2+^ homeostasis (Bai et al. 2009). This suggests that the PERK-based cascade may act by sensing changes in the signal and transduction of the signal to the cell wall during plant growth and development (Nakhamchik et al. 2004). Although previous research has shown that PERK family proteins perform many functions, no information is available on PERK functions in association with low temperature stress.

In the study reported herein, we isolated the *zebra leaf 15* (*z15*) mutant. The *Z15* gene encodes a receptor-like protein kinase of unknown function and belongs to the PERK family. Subcellular localization analysis indicates that Z15 and z15 are localized on the plasma membrane. The gene responds to a moderate low temperature of 18 °C and involves in the moderate low temperature signaling pathway. Mutation of *Z15* disrupts the response to cold stress and cell development in rice. The present results indicated that the PERK performs a novel function in leaf cell development, which provides insights into the underlying mechanism of the response in rice to stress induced by moderate low temperature.

## Results

### The z15 mutant showed the zebra-leaf phenotype and cell development was impaired

**Fig. 1:**
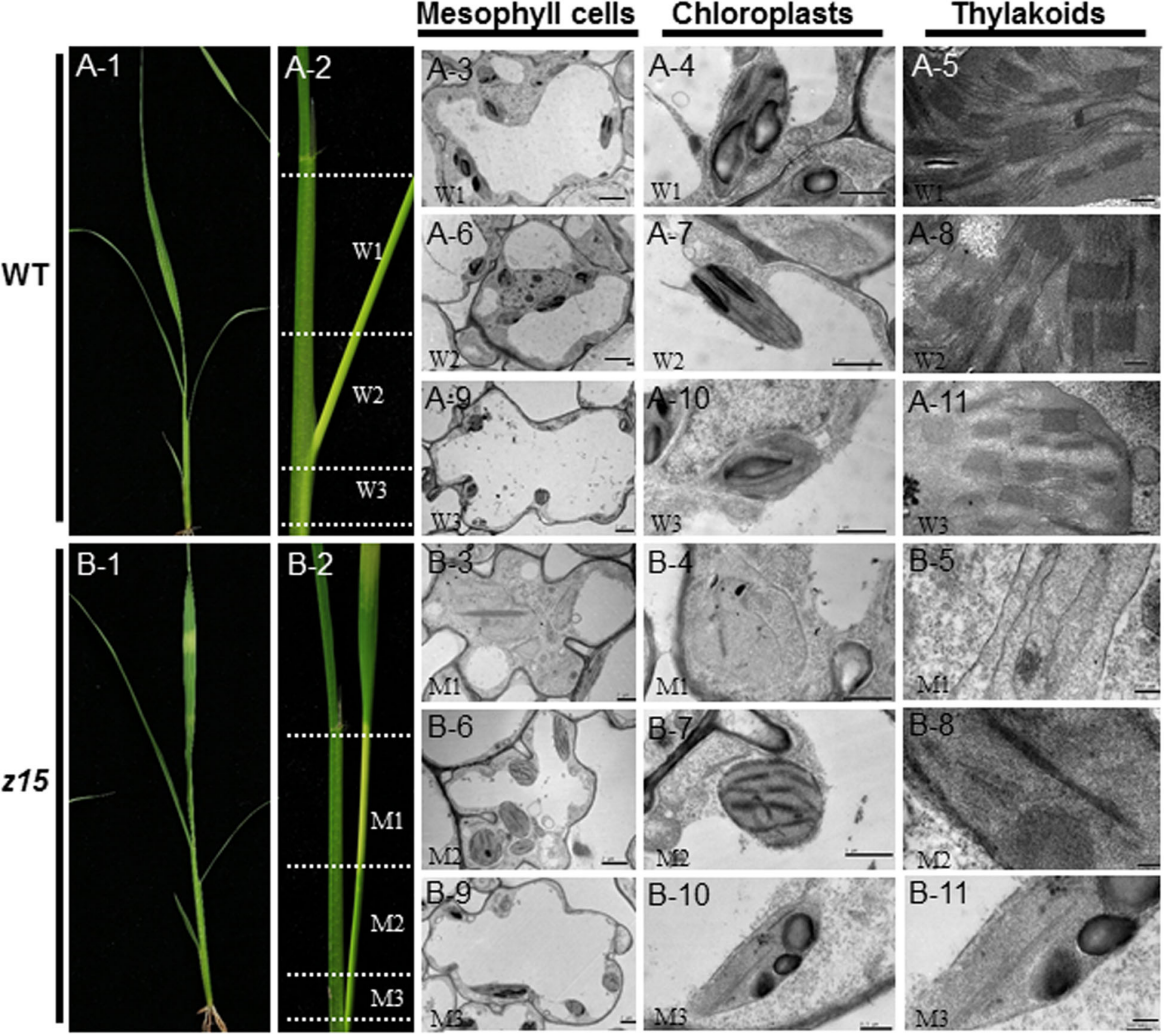
Phenotype of the wild type (WT) and *z15* mutant and transmission electron micrographs of chloroplasts from wrapped leaves. WT (W1, W2, W3), *z15* (M1, M2, M3). A-3, A-6, A-9, B-3, B-6, B-9: bars = 2 μm. A-4, A-7, A-10, B-4, B-7: bars = 1 μm. B-10: bar = 0.5 μm. A-5, A-9, A-11, B-5, B-8, B-11: bars = 200 nm

**Fig. 2:**
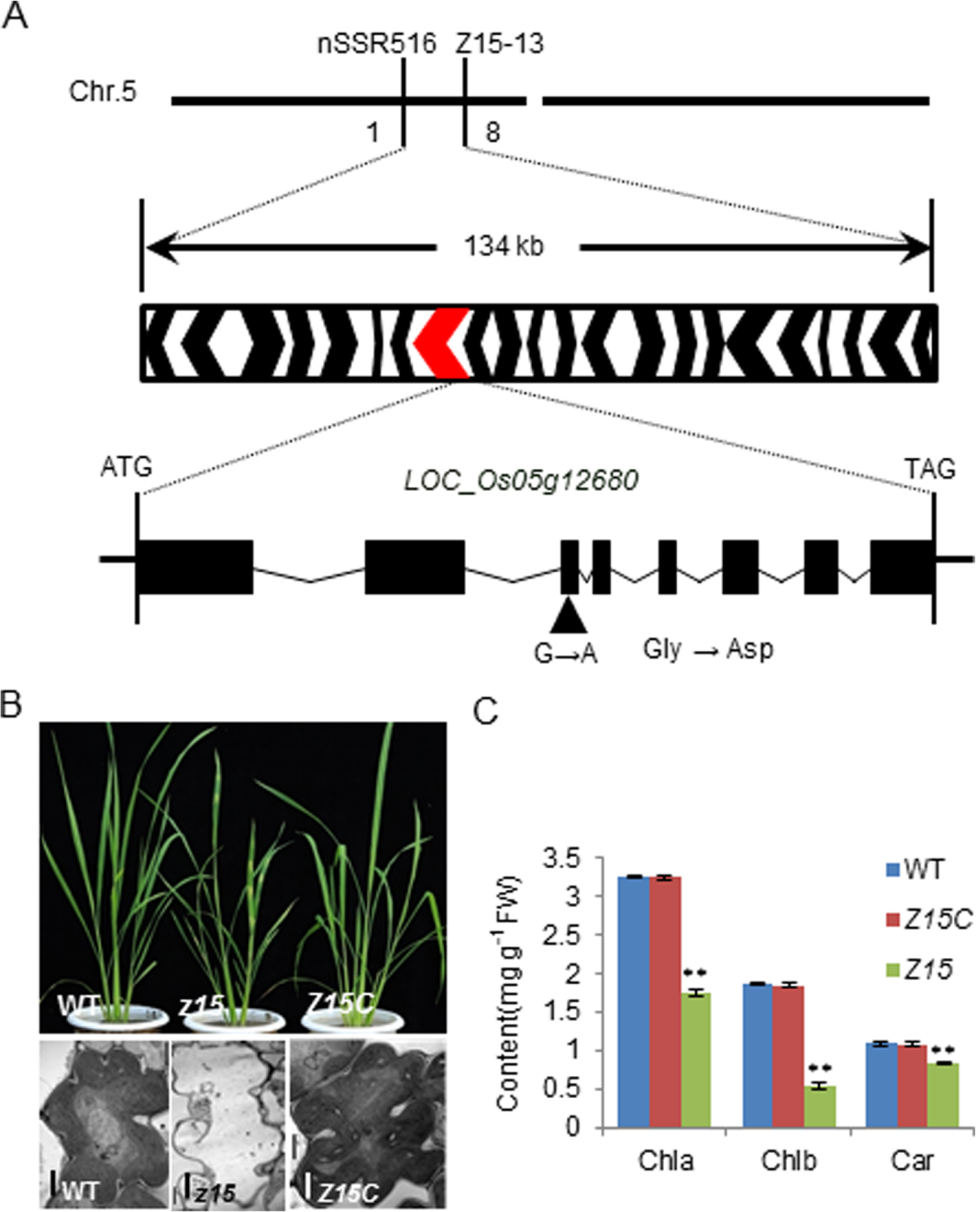
Molecular identity of *Z15*. **a** Map-based cloning of the *Z15* gene. **b** Phenotypes of the wild type (WT), *z15* mutant, complemented transformant (com). **c** Chlorophyll contents of the leaves of the wild type, complemented transformant (Z15C), and z*15* mutant plants at the tiller stage. Bars: (B) =2 μm

Under field conditions, the wild type ‘Jinhui 10’ rice plant developed green leaves throughout the growth period except in the later mature stage (Fig. 1a-1, Additional file 1: Figure S1A). The *z15* mutant plants produced leaves striped transversely with yellow-green or white-green sectors from the seedling stage to the tiller stage, but the leaf sheath showed normal pigmentation (Fig. 1b-1, Additional file 1: Figure S1B). As the leaf developed, the defective phenotype was gradually restored and disappeared after the tiller stage. To determine the timing of appearance of the zebra leaf phenotype in the *z15* mutant, young leaves at seedling stage that had not emerged from the sheath were observed. The zebra leaf phenotype developed before the leaf emerged from the sheath compared with the green leaves in the wild type under the temperature changes from 15 °C to 28 °C (Fig. 1, a-2 and b-2). To investigate whether *Z15* affects leaf chloroplast development, white areas (M1), yellow-green areas (M2), and white areas (M3) of the leaves in the *z15* mutant were observed by transmission electron microscopy (TEM). In the corresponding portions of the wild-type leaf (W1, W2, and W3), all developed complete cells, and the chloroplasts were well developed and well organized in the mesophyll cells. In contrast, in the *z15* mutant the white areas (M1 and M3) contained mostly empty cells and the chloroplasts were impaired, whereas the yellow-green (M2) cells were normal. At the tiller stage, in the wild type, the cells developed normally and contained a complete suite of well-developed organelles (Additional file 1: Figure S2A). In the *z15* mutant, most cells contained a small number of chloroplasts and the structure of some chloroplasts was impaired; however, some cells were empty and the organelles suffered severe damage (Additional file 1: Figure S2B). In addition, we observed that the timing of emergence of the yellow or white portions of the leaves in the *z15* mutant was random by drawing line in the morning and night (Additional file 1: Figure S1B). At the mature stage, the phenotype of the *z15* mutant was identical to that of the wild type with normal green-pigmented leaves (Additional file 1: Figure S1A).

Agronomic traits at maturity were investigated. Compared with those of the wild type, plant height, grain number per panicle, percentage seed set, and 1000-grain weight were significantly reduced in the *z15* mutant, in average by 5.26%, 9.99%, 16.96% and 2.76%, respectively (Additional file 1: Figure S3, A, D, E and F), whereas the number of productive panicles per plant and panicle length showed no significant difference from the wild type (Additional file 1: Figure S3, B and C). These results indicated that the mutation of *Z15* disrupts cell development, thereby impairing chloroplast structure and causing a zebra leaf phenotype under field conditions.

### Molecular cloning and identification of Z15 and subcellular localization of Z15 protein

The *Z15* gene has been mapped to a region of about 268 kb on chromosome 5 (Wang et al. 2009). In our study, the location of *Z15* was narrowed to a physical distance of 134 kb between the insertion/deletion markers nSSR516 and Z15–13. The interval included 23 annotated genes (http://www.gramene.org/). Sequencing analysis identified a signal-nucleotide substitution from G to A within the *LOC_Os05g12680* gene, which caused an amino acid mutation of Gly-409 to Asp-409 (Fig. 2a). To confirm whether the mutation of *LOC_Os05g12680* resulted in the mutant phenotype, we performed a complementation experiment by transforming into the *z15* mutant a 9085-bp wild-type DNA fragment that contained *LOC_Os05g12680*. In the transgenic plants, the phenotype was recovered completely (Fig. 2b). Observation by TEM showed that the mesophyll cells and organelles were similar to those of the wild type (Fig. 2b). In addition, the contents of chlorophylls and carotenoids in the transgenic plants were almost identical to those of the wild type (Fig. 2c), and the contents of chlorophylls and carotenoids in the *z1*5 were significantly lower than that of wild type and the transgenic plants (t-test, *p* < 0.01). Taken together, these results confirmed that *LOC_Os05g12680* corresponded to the *Z15* gene.

**Fig. 3:**
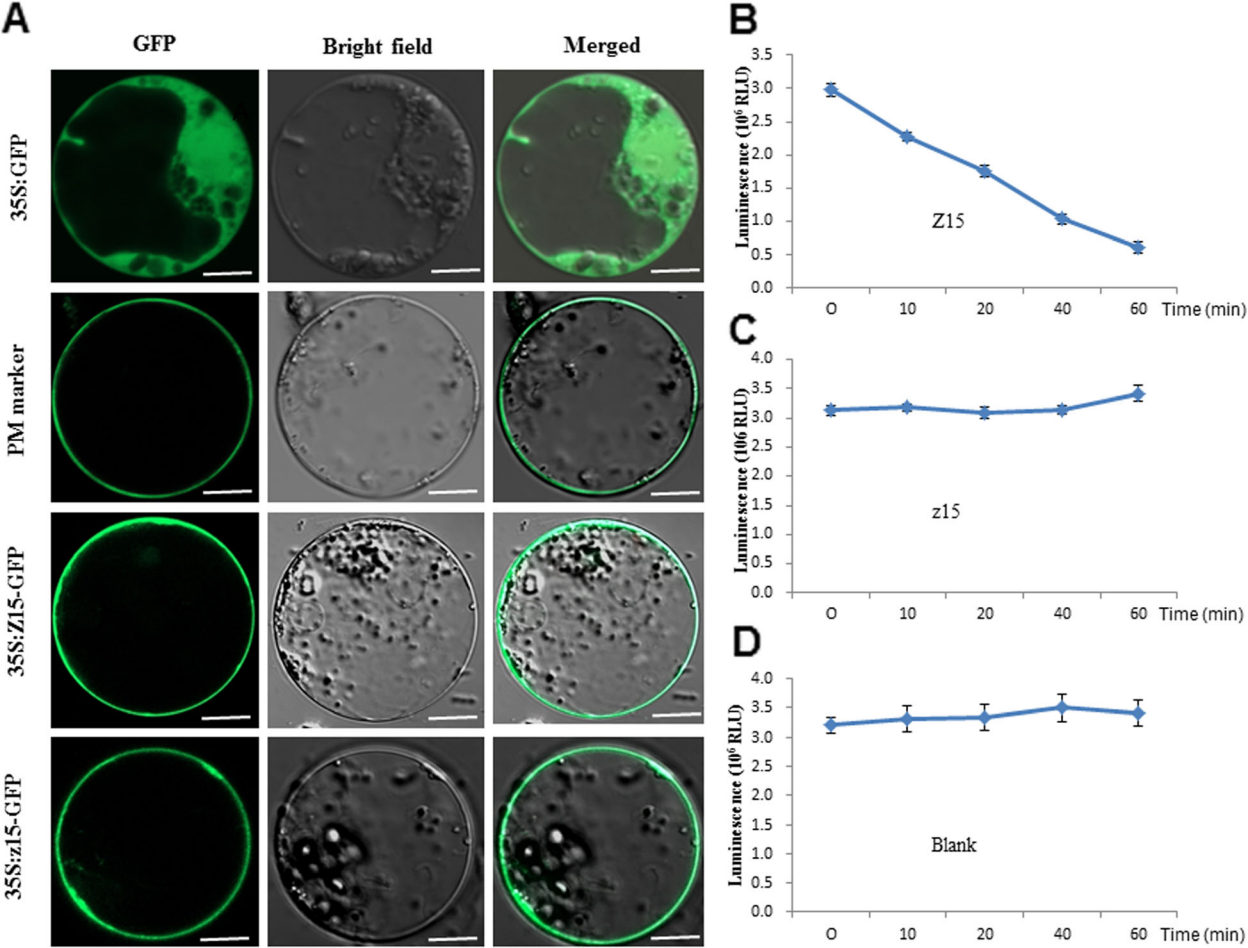
Subcellular localization and kinase activities of Z15 and z15 proteins. **a** Analysis of Z15 and z15 subcellular localizationin rice protoplasts. ESL4-GFP was used as a plasma membrane (PM) marker. Scale bars are 10 μm; **b**-**d**, kinase activity in presence of recombinant Z15 (**b**) or z15 (**c**) and in absence of recombinant protein (**d**). The data are shown as the mean ± SD (*n* = 3)

To examine the subcellular localization of the Z15 and z15 proteins, vectors were constructed to produce fusion proteins with the green fluorescent protein (GFP), Z15-GFP and z15-GFP. In addition, we used ESL4-GFP (Xing et al. 2018) which is located on the plasma membrane, as a positive control. The vectors were introduced into rice protoplasts and the proteins transiently expressed. In cells that expressed GFP alone, green fluorescence was detected consistently throughout the cytosol, while the Z15-GFP and z15-GFP fusion proteins were localized in the cell periphery, similarly to the positive control, consistently with expected plasma membrane localization (Fig. 3a). These results indicate that Z15 is a plasma membrane protein and that the substitution Gly-409 to Asp-409 does not change the protein localization.

### Z15 encodes a receptor-like kinase

**Fig. 4:**
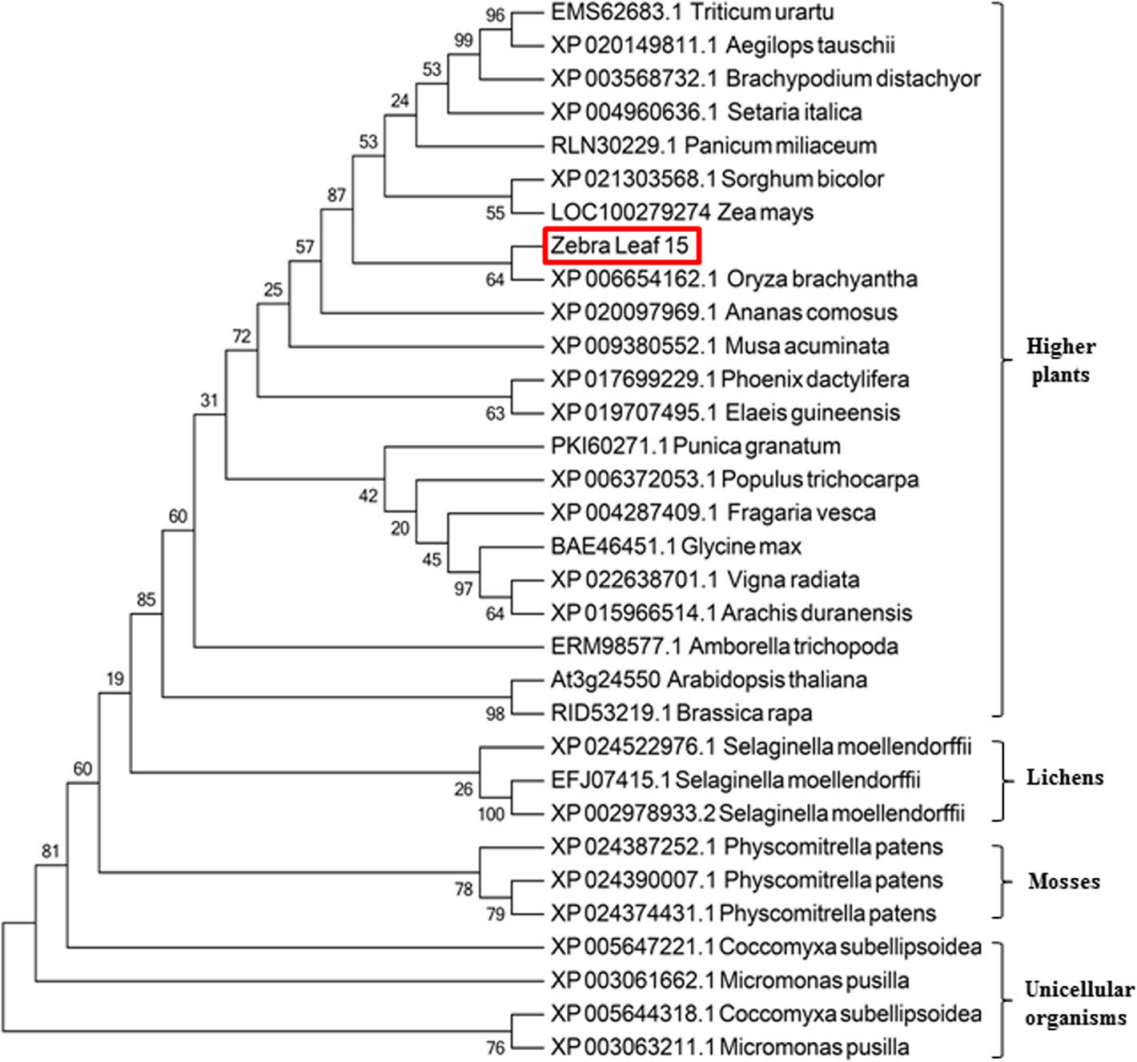
Phylogenetic relationships of the rice Z15 protein. The tree was constructed using the maximum-likelihood method based on the Jones–Taylor–Thornton matrix-based model. Bootstrap support values calculated from 500 replicates are given at the branch nodes. The Z15 protein is highlighted in red

Protein sequences for Z15 were downloaded from the National Centre of Biotechnology Information databases (https://www.ncbi.nlm.nih.gov/) and aligned for phylogenetic analysis. *Z15* encodes a receptor-like protein kinase that belongs to the RLK family. The RLK family is a class of single transmembrane proteins located on the plasma membrane that sense and transmit a variety of signals to regulate plant growth and development (Shiu and Bleecker 2001). The phylogenetic tree indicated that Z15 is highly conserved in many organisms, ranging from unicellular organisms, to mosses and lichens, to higher plants (Fig. 4). In addition, the Z15 protein cluster showed the highest similarity to the PERK-like receptor protein kinase family. The conserved domain of the protein sequence contains a STKc_IRAK domain, which is a catalytic domain of Ser/Thr kinases (Additional file 1: Figure S4). We performed a predictive analysis of the kinase domain and observed that the proton acceptor is close to the mutation site (Additional file 1: Figure S5, A, B and C). The mutation of *Z15* caused an amino acid mutation of Gly to Asp (Fig. 2a), i.e., substitution of a neutral amino acid with an acidic amino acid, which may be due to the changes in kinase activity. So we performed an in vitro enzyme activity assay to determine whether Z15 and z15 have kinase activities. We constructed pet32a vectors to express proteins containing the Z15 and z15 intracellular domains. The kinase activity of the recombinant proteins were determined by measuring ATP consumption in presence of a classical kinase substrate, the myelin basic protein (MBP) and using a luminescent assay kit. In presence of Z15, the relative light unit measured decreased over time (Fig. 3b), which indicates that ATP was continuously consumed during the enzymatic reaction and that the Z15 intracellular domain is catalytically active. In contrast, in presence of the z15 intracellular domain, the relative light unit was almost unchanged (Fig. 3c), which indicates that mutation of Z15 results in a loss of kinase activity.

### Expression pattern of Z15

**Fig. 5:**
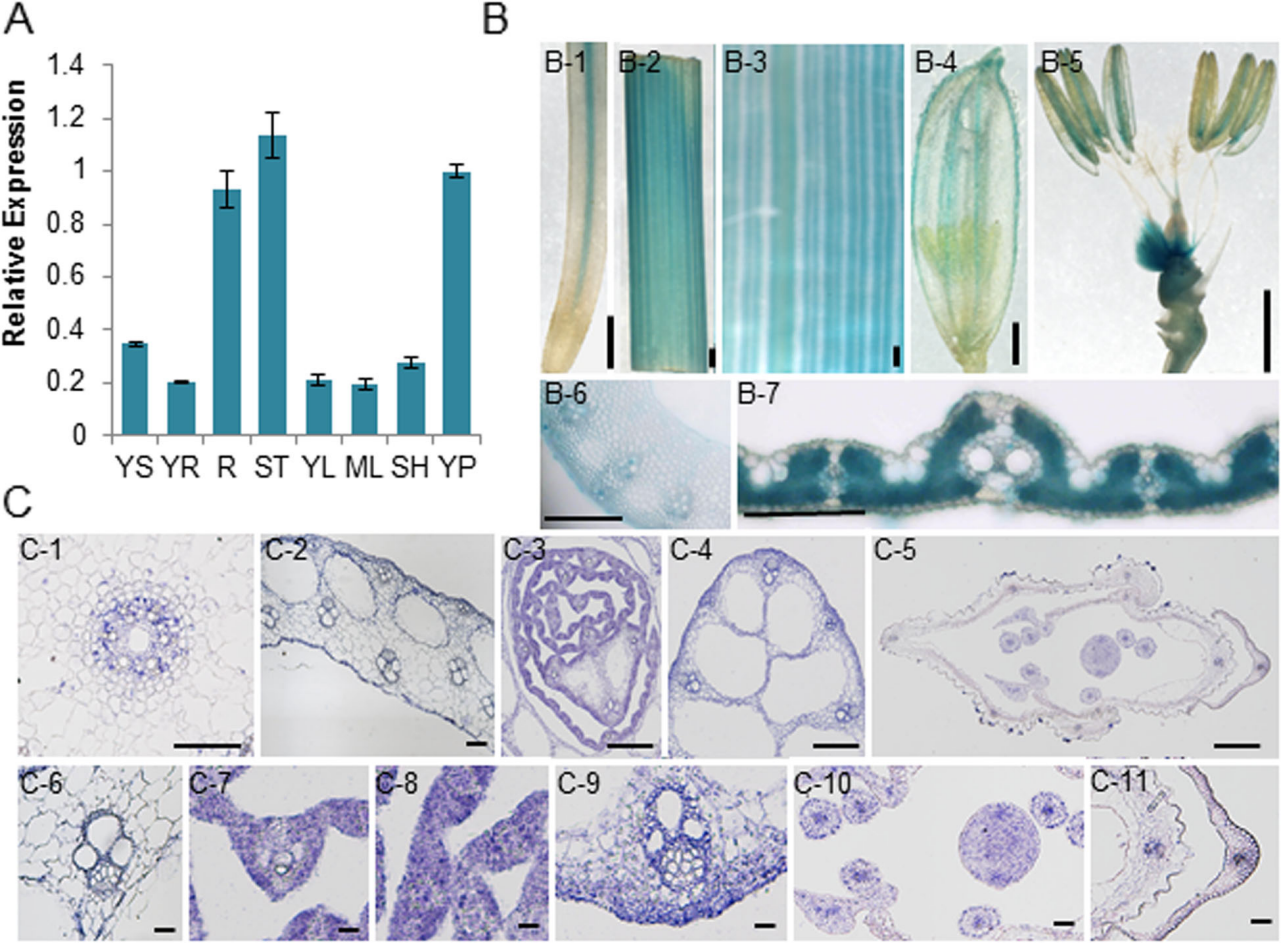
Expression patterns of *Z15.* **a** Expression patterns of *Z15* of the wild type in different tissues as indicated by quantitative real-time PCR. YS: leaf at seedling stage, YR: root at seedling stage; R: root, ST: stem, YL: young leaf, ML: mature leaf, SH: sheath, YP: young panicle. **b** GUS expression in various tissues of ProZebra15::GUS transgenic plants (B-1: root, B-2: stem, B-3: leaf, B-4: panicle, B-5: stamen, B-6 and B-7: hand cross-sections of stem and leaf. (B-1 to B-6: bar = 1 mm, B-7: bar = 25 μm), **c** Expression patterns of *Z15* indicated by in situ hybridization (C-1: root, C-2: stem, C-3: leaf, C-4: sheath, C-5: panicle, C-6 is an enlarged view of C-2, C-7 and C-8 are enlarged view of C-3, C-9 is an enlarged view of C-4, C-10 and C-11 are enlarged view of C-5, C-6 to C-11: bar = 100 μm)

Quantitative real-time PCR (qPCR) analysis revealed that *Z15* was expressed in a variety of tissues of the wild type, including the root, stem, young leaf, mature leaf, sheath, and young panicle (Fig. 5a).

**Fig. 6:**
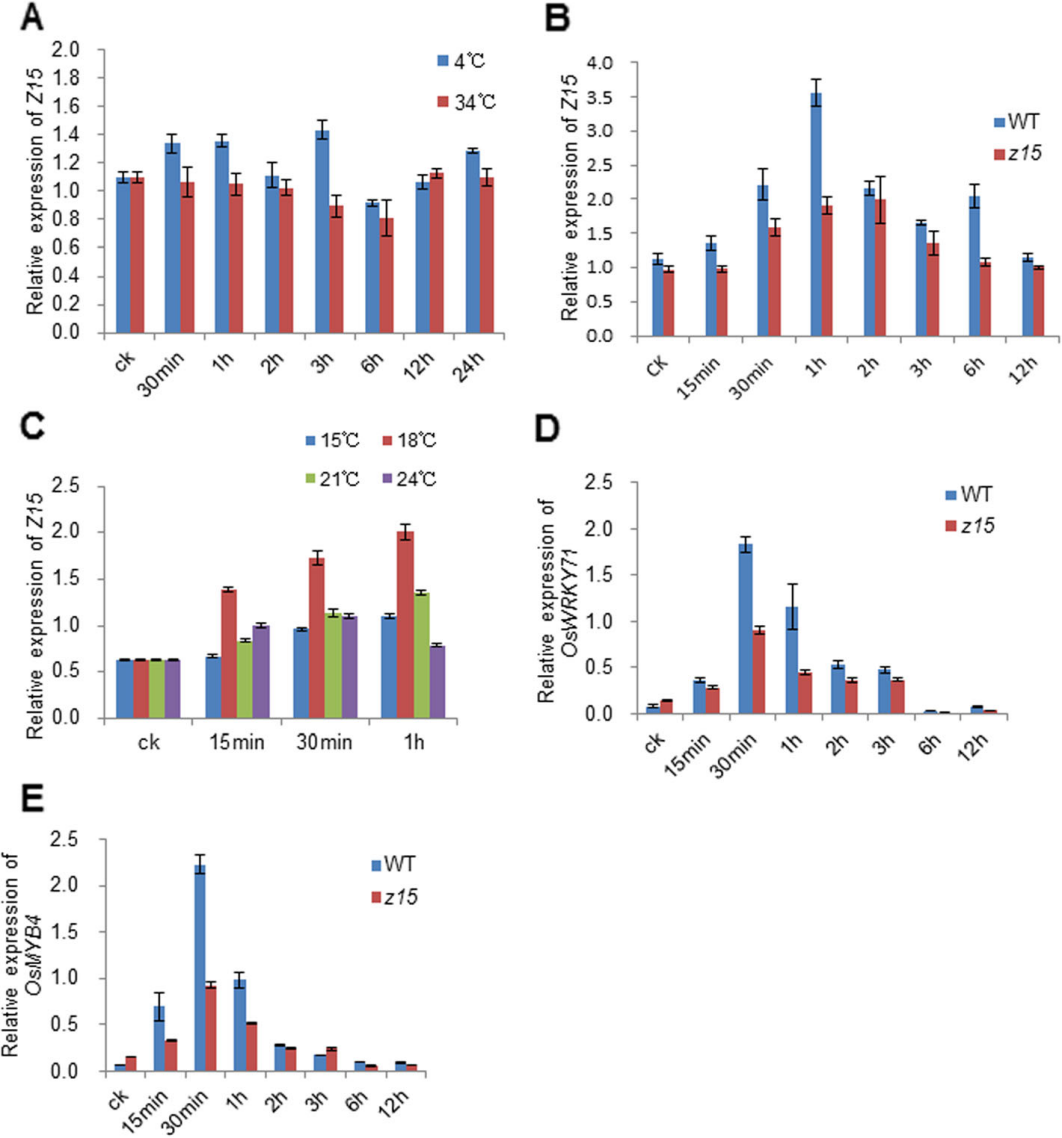
Influence of temperature on expression of *Z15.* **a** Expression of *Z15* at 4 °C and 34 °C, respectively. **b** Expression of *Z15* in wild type and *z15* mutant at 18 °C. **c** Expression of *Z15* at 15, 18, 21, and 24 °C, respectively. **d** The expression of *OsWRKY71* at 18 °C. **e** The expression of *OsMYB4* at 18 °C*.* Error bars represent the standard deviation (SD) of three biological repeats

To gather further information on *Z15* expression, a binary vector containing the *Z15* and β-glucuronidase (GUS) genes driven by the *Z15* promoter (*Z15*::*GUS*) was constructed. GUS expression was detected in the root, stem, leaf, and panicle (Fig. 5b), whereas *Z15* was expressed predominantly in vascular bundles in the root, panicle, and stamen (Fig. 5, b-1, b-4, and b-5). Histochemical localization revealed that GUS expression was restricted exclusively to vascular bundles and epidermal cells in the stem and mesophyll cells in leaf (Fig. 5, b-6 and b-7).

For a more detailed analysis of the expression pattern of *Z15*, transverse sections of various tissues were examined for *Z15* signals by in situ hybridization (Fig. 5c). Consistent with the GUS expression results, the *Z15* signal was observed in the vascular bundle of the root (Fig. 5c-1). In the stem, sheath, and young panicle, signals were detected in the vascular bundles and epidermal cells (Fig. 5c-2, c-6, c-4, c-9, c-5, c-10, and c-11). Interestingly, *Z15* signals were concentrated mainly in mesophyll cells in young leaves (Fig. 5c-7 and c-8).

### Z15 is involved in moderate low temperature signaling pathway

As the phynotype of *z15* was expressed under field conditions, we examined the expression of *Z15* under different temperatures by qPCR. The expression of *Z15* was not affected significantly under high temperature (34 °C) and low temperature (4 °C) from 30 min to 24 h, compared with the control under 28 °C (Fig. 6a). However, when treated at 18 °C, expression of *Z15* was induced distinctly at 1 h (Fig. 6b), and thereafter the level of expression of *Z15* fell gradually and stabilized. For a more detailed examination of the response of *Z15* to temperature, seedlings were treated at 15, 18, 21, and 24 °C, respectively. The highest level of expression of *Z15* was observed at 18 °C (Fig. 6c). We have also analyzed the expression of *Z15* in *z15* mutant under 18 °C. We found that the expression of *Z15* was less induced in the *z15* mutant compared to the wild type (Fig. 6b). Taken together, these results indicated that the expression of *Z15* was induced by moderate low temperature (18 °C), rather than a high temperature (34 °C) or low temperature (4 °C).

To obtain insights into the function of *Z15*, the transcriptomic profiles of seedlings of the *z15* mutant and wild type treated at 18 °C for 30 min were characterized. Comparisons of the differentially expressed genes (DEGs) of the different treatment groups, namely *z15* (30 min) vs *z15* (CK) and the wild type (30 min) vs the wild type (CK), are presented (Additional file 1: Figure S6, B and C). The two comparisons shared 2038 DEGs (Additional file 1: Figure S6A), which indicated that these common DEGs continued to function during 30 min of cold-stress treatment. Among the common 2038 DEGs, the expression of certain genes involved in the cold-stress response changed significantly, which indicates that cold tolerance was affected strongly by the mutation of *Z15* (Additional file 2: Tables S2 and S3). Previous studies indicated that *OsWRKY71* and *OsMYB4* were induced by cold stress specifically (Kim et al. 2016; Vannini et al. 2004). In our study, the expression of these two genes increased significantly after 6 h of 4 °C treatment (Additional file 1: Figure. S6, D and E), and the genes were expressed at an early stage of 18 °C stress (Fig. 6d and e). These results indicated that *OsWRKY71* and *OsMYB4* are responsive not only to 4 °C, but also to 18 °C. In addition, qPCR verified that the levels of expression of *OsWRKY71* and *OsMYB4* were lower in the *z15* mutant than in the wild type (Fig. 6d and e), which indicated that *Z15* influenced the expression of *OsWRKY71* and *OsMYB4*. Taken together, the results suggested that *Z15* is involved in moderate low temperature signaling pathway in rice.

## Discussion

We characterized a novel rice zebra leaf mutant, *z15*, which showed transversely striped leaves with yellow-green and white-green sectors in which chloroplast structure is impaired. In addition, observation by TEM showed that the structure of a variety of organelles was damaged in the yellow or white areas of leaves (Fig. 1, Additional file 1: Figure S1). *Z15* was determined to encode a RLK protein. The RLK family members play important roles in mediating the cell response to a variety of environmental cues. The expression of *Z15* is induced by moderate low temperature (18 °C). The mutation of *Z15* influenced not only its own expression at moderate low temperature, but also the expression of downstream genes that respond to moderate low temperature. The present results showed that *Z15* is involved in moderate low temperature signaling pathway, which plays a crucial role in the early response of rice to moderate low temperature.

In previous studies, a number of genes associated with the zebra leaf phenotype in rice were cloned. The *zebra necrosis* (*zn*) mutant produces transversely striped leaves with green and yellow sectors, and necrotic lesions often develop in the yellow sectors during leaf elongation. *ZN* encodes a thylakoid-bound protein of unknown function. The necrotic lesions in yellow sectors of *zn* mutant leaves develop as a result of excessive accumulation of reactive oxygen species (Li et al. 2010). The *zebra leaf 2* (*z2*) mutant shows alternating transverse green and yellow sectors on the leaves, and the phenotype is affected by temperature and light. *ZEBRA2* encodes a carotenoid isomerase, the key enzyme that catalyzes the conversion of *cis*-lycopene to all-*trans*-lycopene. A more severe phenotype of the *zebra2* mutant manifests under high light intensity, which indicates that carotenoids biosynthesis is impaired because of the gene mutation, and thus causes photooxidation and ABA defects, and leads to the zebra-leaf phenotype (Chai et al. 2011). Additional studies suggest that leaf variegation in the *zebra2* mutant is caused by photoperiodic accumulation of tetra-*cis*-lycopene and singlet oxygen (Han et al. 2012). The rice *zebra 3* (*z3*) mutant shows transverse dark-green or green sectors in mature leaves and lacks the typical yellow or white sectors. *Z3* encodes a putative citrate transporter that belongs to the citrate-metal hydrogen symport family. The *z3* phenotype is caused by unbalanced accumulation of citrate in transverse sectors of the leaf (Kim et al. 2018). The rice *zebra leaf 16* (*z16*) phenotype is light-dependent and is expressed as chlorotic abnormalities in transverse sectors of the young leaves of seedlings. *Z16* encodes a β-hydroxyacyl-ACP dehydratase involved in de novo fatty acid synthesis. The *z16* mutation affects early stages of chloroplast development in leaves (Liu et al. 2018). In our study, plants of the *z15* mutant produced transversely striped leaves with yellow-green or white-green sectors from the seedling stage to the tiller stage (Fig. 1b-1, Additional file 1: Figure S2 B-2), but the leaf sheath showed normal pigment development. Under field conditions, the phenotype of *z15* is sensitive to cold stress. In contrast to the *zn* mutant, no necrotic lesions develop on the leaves of the *z15* mutant, and unlike the *z2* mutant, the phenotype of *z15* is unaffected by light intensity. With regard to subcellular structure, unlike the *zn* and *z2* mutants, a variety of organelles were damaged in addition to plastids and chloroplasts (Fig. 1, Additional file 1: Figure S1). Therefore, *z15* is a novel zebra leaf mutant. *Z15* encodes a receptor-like protein kinase, which is distinct from *ZN* and *Z2*, thus *Z15* is a newly identified gene. We suggest that *Z15* is a novel regulatory factor that affects leaf development indirectly by regulating signalling in response to moderate low temperature.

It has been reported that RLKs are involved in plant response to low-temperature stress and transmit low-temperature signals (Liu et al. 2017). The PERK family (proline-rich, extension-like receptor kinases) belongs to the RLKs family. *Z15* encodes a receptor-like protein kinase of unknown function, which shows homology with the PERK family (Fig. 4). Information on PERK receptor protein kinases is limited, but previous studies have shown that PERK-based signal cascades may act by sensing signal changes and transduction of signals to the cell wall during plant growth and development (Nakhamchik et al. 2004). In *Brassica napus*, BnPERK1 may be involved in the plant’s early defence responses to mechanical stress and fungal attack (Silva and Goring 2002). *AtPERK4*, which encodes a receptor-like kinase, plays a role in the early stages of ABA signalling and inhibits root cell elongation by disrupting Ca^2+^ homeostasis (Bai et al. 2009). In the study reported herein, we provide evidence that *Z15* is involved in moderate low temperature signaling pathway. First, we showed that the zebra leaf phenotype of the *z15* mutant manifested only under field conditions, and the time of emergence of the yellow or white sectors of the leaf was random (Additional file 1: Figure S1). Second, we observed that *Z15* was less induced in *z15* mutant than wild-type by moderate low temperature (18 °C) (Fig. 6b). Third, *Z15* encodes a receptor-like kinase**,** which localizes at the plasma membrane. The mutation in z15 protein doesn’t affect the plasma membrane localization but abolishes the in vitro kinase activity (Fig. 3aand c). In addition, *Z15* was expressed predominantly in vascular bundles in the root, stem, sheath, and panicle, and in mesophyll cells in the leaf (Fig. 5). Thus, a novel function of PERK family proteins was revealed that Z15 is involved in moderate low temperature signaling pathway. Finally, we identified two potential downstream cold-defence genes, namely *OsWRKY71* and *OsMYB4.* In rice, overexpression of *OsWRKY71* in rice results in increased resistance to disease (Liu et al. 2007). *OsWRKY71* is also induced by cold stress (Kim et al. 2016). Similarly, our study showed that the *OsWRKY71* expression level was decreased in the *z15* mutant after treatment with moderate low temperature, compared with the wild type (Fig. 6d). Previous research has demonstrated the ability of *OsMYB4* to positively regulate the transcription of genes involved in the cold response (Vannini et al. 2004). In Arabidopsis, *AtMYB14* is down regulated under cold treatment and its involvement in freezing tolerance is affected by the expression of CBF genes (Chen et al. 2013). Interestingly, the expression of *OsMYB4* is consistent with *OsWRKY71* in response to cold stress (Fig. 6e). Thus, the above-mentioned results indicated that *Z15* may regulate the expression of the potential downstream cold-response genes *OsWRKY71* and *OsMYB4*. Therefore, expression of the Z15 receptor kinase is indicated to be an early response to exposure to moderate low temperature.

The mechanism by which *Z15* is involved in moderate low temperature signaling pathway was investigated. The present observations suggest that *Z15* might play an important role in early stages of the response to exposure to moderate low temperature.

## Conclusion

A novel zebra-leaf mutant was identified in rice, *LOC_Os05g12680*, encoding a receptor-like protein kinase both of Z15 and z15 were located on plasma membrane, but the mutation of Z15 resulted in loss of kinase activity. The expression of *Z15* was optimally induced by moderate low temperature, under which condition the transcriptional regulon of *Z15* are mainly involved in response to low temperature (4 °C). Among of them, the expression of two selected *Z15*-affecting genes was specifically induced only at the temperatures ranging from low to moderate low, confirming our results that *Z15* is involved in moderate low temperature signaling pathway, which plays a crucial role in the early stage of freezing tolerance in rice growth. Results from this work suggest that *Z15* is involved in moderate low temperature pathway, which plays a crucial role in the early stages of the response to moderate low temperature in rice.

## Materials and methods

### Plant materials

The *z15* mutant was derived from rice (*Oryza sativa L. subsp*. *indica*) ‘Jinhui 10’ treated with ethyl methanesulfonate. ‘Jinhui 10’ was used as the wild type in all experiments. All plants were grown in an experimental field at Chongqing, China, under natural conditions. We cultivated seeds with nutrient solution. ‘Jinhui 10’ seeds were cultured in a light incubator (RXZ-500) under 12:12 h light (400 μm/m^2^/s)/darkness cycle at temperature of 28 °C (day and night). For temperature treatment, we set the temperature in advance, and put the same nutrient solution into the incubator so that the water temperature is the same as the incubator temperature.

### Photosynthetic pigments analysis

Photosynthetic pigments were extracted from equal fresh weight (~ 0.1 g) of mature leaves from the wild-type, *z15*, and transgenic plants in 25 ml extraction buffer (ethanol:acetone, 1:1; v/v) for 24 h at room temperature in the dark. The concentrations of chlorophylls and carotenoids were determined with a UV-1800PC spectrophotometer (Mapada Co. Ltd., Shanghai, China) in accordance with the method of (Lichtenthaler 1987).

### Agronomic trait analysis

Agronomic traits (comprising plant height, number of productive panicles per plant, panicle length, grain number per panicle, percentage seed set, and 1000-grain weight) for each of the *z15* mutant and the wild type were analyzed at the mature stage with 15–20 replicates.

### Transmission electron microscopy

Leaves of wild-type, *z15*, and transgenic plants were investigated by TEM using the method described by (Zhu et al. 2016), The middle portion of the leaves of the plants grown in the experimental field under natural conditions were collected and fixed in a primary fixative solution (3.5% glutaraldehyde) for 2 d at room temperature. Each sample was washed with 0.1 mol l^− 1^ phosphate buffer solution, then post fixed for 2 h with 1% osmium tetroxide. The tissues were then stained with uranyl acetate, dehydrated in ethanol, and embedded in Spurr’s resin before ultrathin sectioning. Each sample was then restained and examined using a H-7500 transmission electron microscope (Hitachi High-Technologies).

### Fine-mapping of Z15

The *z15* mutant was crossed with ‘Xinong1A’ (bred by the Southwest University Rice Research Institute, Chongqing, China) to obtain the F_1_ population, and the F_2_ population was generated by self-fertilization of F_1_ plants. The F_2_ plants that manifested the mutant phenotype were selected and used to map the genomic location of *Z15*. Fine-mapping was performed using insertion/deletion markers that were developed from comparison of genomic sequences for ‘Xinong1A’ and ‘Jinhui 10’ in our laboratory. The sequences of primers used in fine-mapping are listed in Additional file 2: Table S1.

#### Method for verifying the complementation of the gene

Within the physical distance of 134 kb in our mapped location of *Z15*, we have sequenced the exon region of all the genes (23 in total), identified a single nucleotide substitution on the third exon of *LOC_Os05g12680* in the *z15* mutant compared to the wild-type (Fig. 2a). Therefore, we regarded *LOC_Os05g12680* as a candidate gene. We then performed a complementation experiment by transforming the *z15* mutant with a T-DNA construct containing a 9085-bp *Z15* genomic DNA fragment (*LOC_Os05g12680*, including 2978-bp of the promoter), neighbored by an hygromycin resistance cassette and the GUS reporter gene under the control of the 35S promoter (Additional file 1: Figure S7). We obtained six transgenic plants. The method for determining full insertion of the T-DNA was firstly sequencing plant growth on hygromycin and PCR amplification of the hygromycin resistance gene. And secondly, GUS staining. We then observed the phenotype of T_0_ and T_1_ transgenic plants, and their phenotype was stable. Finally, we have used the primers F1 and R1 (Additional file 1: Figure S7) amplify and sequence the endogenous Z15 allele and verify that the transgenic plants were on the homozygous mutant background. We also used the primers F2 and R1 to verify that the WT copy was also present in the transgenic plants. By transmission electron microscopy and chlorophylls and carotenoids content determination, we determined that the phenotypes of these six positive transgenic plants were similar to those of the wild type (Fig. 2b). Taken together, this shows that the mutation in *LOC_Os05g12680* is responsible for the observed phenotype in the *z15* mutant.

### Vector construction for genetic complementation and ProZ15::GUS

To construct the *Z15* complementation plasmid, a 9085-bp genomic fragment containing the *Z15* coding sequence, coupled with 3022-bp upstream and 1224-bp downstream sequences, was cloned into the binary vector pCAMBIA1301. To analyze the expression pattern of *Z15*, the Pro*Z15*::GUS vector was constructed by amplifying the upstream 3026-bp promoter sequence of *Z15* from ‘Jinhui 10’ genomic DNA, then cloning the sequence into the pCAMBIA1301 vector. The recombinant complementation plasmids were introduced into *z15* using the *Agrobacterium tumefaciens*-mediated transformation method as described previously (Zhang et al. 2017). The primer pairs used for vector construction are listed in Additional file 2: Table S1.

### RNA extraction and quantitative real-time PCR analysis

Total rice RNA was extracted from the roots, stems, leaves, leaf sheaths, and young panicles using the RNAprep Pure Plant Kit (Promega Co., Ltd., Beijing, China). All reverse transcriptions were performed using the GoScript™ Reverse Transcription System (Promega). After cDNA synthesis, all samples were diluted 10 times and stored at − 20 °C. Quantitative RT-PCR was performed using TB Green™ Premix Ex Taq™ II (Tli RNaseH Plus) (TaKaRa, China) in a Bio-Rad Real-Time System (1000 Alfred Nobel Drive, Hercules, California 94,547, U.S.A). Three replicates were performed and the relative level of expression of each gene was calculated using the 2^−△△*Ct*^ method and expressed relative to *OsActin* expression. The primers used are listed in Additional file 2: Table S1.

### Subcellular localization

The full-length coding sequence of *Z15* and *z15* without the stop codon were fused to the N-terminus of the *GFP* gene under the control of the enhanced *Cauliflower mosaic virus 35S* promoter in the *Spe*I and *Bam*HI sites of the vector pAN580 to generate the pAN580-*Z15*-*GFP* and pAN580-*z15*-*GFP* construct. The vectors were introduced into rice protoplasts using a method described previously (Ma et al. 2017). The GFP fluorescence was determined using an LSM710 confocal laser scanning microscope (Zeiss, Jena, Germany). The primer sequences are listed in Additional file 2: Table S1.

#### Determining the activities of Z15 and z15 kinase

Pet32a vectors allowing expression of Z15 and z15 intracellular domains (from 181th amino acid to 675th amino acid) tagged with 6X His in *Escherichia coli* were built. The primer sequences used for cloning are listed in Additional file 2: Table S1.

The recombinant proteins expressed in the *E.coli* (*DE3*) were purified using Beaver Beads™ IDA-Nickel kit (cat70501-klo; Haitian Nano Technology Co, Suzhou, China).

Recombinant protein enzymatic activities were performed using a Kinase-Glo R Luminescent Kinase Assay kit (cat no.#M2295; Promega Biotechnology Co., Beijing, China). Enzymatic buffer and the substrate myelin basic protein (MBP) were added to start the reaction. Luminescence was measured as described in (Xing et al. 2018). The blank corresponds to the same reaction mixture (Enzymatic buffer and MBP), in absence of recombinant protein.

### In situ hybridization

The 429-bp gene-specific *Z15* probe was amplified and labelled using a DIG RNA Labeling Kit (SP6/SP7; Roche). Section pretreatment, hybridization, and immunological detection methods were performed using previously described methods (Zhang et al. 2017). The sequences of the primers used are listed in Additional file 2: Table S1.

### Transcriptome analysis

Leaves from 10-day-old plants of *z15* and wild-type grown in an incubator at 28 °C and treated at 18 °C for 30 min were collected for RNA extraction and transcriptome sequencing with three biological replicates. The purity and concentration of RNA were measured using a NanoPhotometer spectrophotometer (Implen, Westlake Village, CA, USA) and the Qubit® RNA Assay Kit in a Qubit® 2.0 Fluorometer (Life Technologies, Carlsbad, CA, USA). A cDNA library was constructed and RNA sequencing (RNA-seq) was performed by the Novogene Bioinformatics Institute (Beijing, China) using a HiSeq 4000 system (Illumina, San Diego, CA, USA). The RNA-seq short reads were aligned to the *indica* rice genome with HISAT2 (https://ccb.jhu.edu/software/hisat2/index.shtml). The level of expression of each gene was computed using StringTie (https://ccb.jhu.edu/software/stringtie/).

## Supplementary information

**Additional file 1: Figure S1.** The phenotype of wild type and *z15* mutant. **Figure S2.** Transmission electron micrographs of chloroplasts at tiller stage. **Figure S3.** Agronomic traits of the wild type (‘Jinhui 10’) and *z15* mutant at mature stage. **Figure S4.** Protein sequence alignment of Z15. **Figure S5.** Protein kinases domain profile. **Figure S6.** Transcriptome analysis and expression of genes involved in cold stress response. **Figure S7.** Sequencing method.

**Additional file 2: Table S1.** Primers used in the study. **Table S2.** DEGs annotated within the cold-response. **Table S3.** DEGs annotated within the cold-response.

## Data Availability

The data sets supporting the conclusions of this article are included within the article and its additional files.

## Abbreviations

CBF: CCAAT motif-binding factor
GFP: Green fluorescent protein
GUS: β-glucuronidase.
PERKs: Proline extension receptors
qPCR: Quantitative real-time PCR
RLKs: Receptor-like protein kinases
TEM: Transmission electron microscopy

## References

[CR1] Bai L, Zhang GZ, Zhou Y, Zhang ZP, Wang W, Du YY, Wu ZY, Song CP (2009). Plasma membrane-associated proline-rich extensin-like receptor kinase 4, a novel regulator of Ca2+ signalling, is required for abscisic acid responses in Arabidopsis thaliana. Plant J.

[CR2] Chai CL, Fang J, Liu Y, Tong HN, Gong YQ, Wang YQ, Liu M, Wang YP, Qian QA, Cheng ZK, Chu CC (2011). ZEBRA2, encoding a carotenoid isomerase, is involved in photoprotection in rice. Plant Mol Biol.

[CR3] Chen Y, Chen ZL, Kang JQ, Kang DM, Gu HY, Qin GJ (2013). AtMYB14 regulates cold tolerance in Arabidopsis. Plant Mol Biol Rep.

[CR4] Dai XY, Xu YY, Ma QB, Xu WY, Wang T, Xue YB, Chong K (2007). Overexpression of an R1R2R3 MYB gene, OsMYB3R-2, increases tolerance to freezing, drought, and salt stress in transgenic Arabidopsis. Plant Physiol.

[CR5] Ding YL, Li H, Zhang XY, Xie Q, Gong ZZ, Yang SH (2015). OST1 kinase modulates freezing tolerance by enhancing ICE1 stability in Arabidopsis. Dev Cell.

[CR6] Dong YJ, Dong WQ, Shi SY, Jin QS (2001). Identification and genetic analysis of a thermo-sensitive seedling-colour mutant in rice (Oryza sativa L.). Breeding Sci.

[CR7] Han SH, Sakuraba Y, Koh HJ, Paek NC (2012). Leaf variegation in the rice zebra2 mutant is caused by photoperiodic accumulation of tetra-cis-lycopene and singlet oxygen. Mol Cells.

[CR8] Kim CY, Vo KTX, Nguyen CD, Jeong DH, Lee SK, Kumar M, Kim SR, Park SH, Kim JK, Jeon JS (2016). Functional analysis of a cold-responsive rice WRKY gene, OsWRKY71. Plant Biotechnol Rep.

[CR9] Kim SH, Kwon CT, Song G, Koh HJ, An G, Paek NC (2018) The rice zebra3 (z3) mutation disrupts citrate distribution and produces transverse dark-green/green variegation in mature leaves. Rice 11(1):1 10.1186/s12284-017-0196-8 PMC5756232 29305728

[CR10] Li H, Ye KY, Shi YT, Cheng JK, Zhang XY, Yang SH (2017). BZR1 positively regulates freezing tolerance via CBF-dependent and CBF-independent pathways in Arabidopsis. Mol Plant.

[CR11] Li J, Pandeya D, Nath K, Zulfugarov IS, Yoo SC, Zhang HT, Yoo JH, Cho SH, Koh HJ, Kim DS, Seo HS, Kang BC, Lee CH, Paek NC (2010). ZEBRA-NECROSIS, a thylakoid-bound protein, is critical for the photoprotection of developing chloroplasts during early leaf development. Plant J.

[CR12] Lichtenthaler HK (1987). Chlorophylls and carotenoids - pigments of photosynthetic biomembranes. Methods Enzymol.

[CR13] Liu XQ, Bai XQ, Wang XJ, Chu CC (2007). OsWRKY71, a rice transcription factor, is involved in rice defense response. J Plant Physiol.

[CR14] Liu ZW, Wang ZY, Gu H, You J, Hu MM, Zhang YJ, Zhu Z, Wang YH, Liu SJ, Chen LM, Liu X, Tian YL, Zhou SR, Jiang L, Liu LL, Wan JM (2018) Identification and phenotypic characterization of ZEBRA LEAF16 encoding a beta-Hydroxyacyl-ACP dehydratase in Rice. Front Plant Sci 9:782 10.3389/fpls.2018.00782 PMC6005893 29946330

[CR15] Liu Ziyan, Jia Yuxin, Ding Yanglin, Shi Yiting, Li Zhen, Guo Yan, Gong Zhizhong, Yang Shuhua (2017). Plasma Membrane CRPK1-Mediated Phosphorylation of 14-3-3 Proteins Induces Their Nuclear Import to Fine-Tune CBF Signaling during Cold Response. Molecular Cell.

[CR16] Ma L, Sang XC, Zhang T, Yu ZY, Li YF, Zhao FM, Wang ZW, Wang YT, Yu P, Wang N, Zhang CW, Ling YH, Yang ZL, He GH (2017). ABNORMAL VASCULAR BUNDLES regulates cell proliferation and procambium cell establishment during aerial organ development in rice. New Phytol.

[CR17] Ma Yun, Dai Xiaoyan, Xu Yunyuan, Luo Wei, Zheng Xiaoming, Zeng Dali, Pan Yajun, Lin Xiaoli, Liu Huanhuan, Zhang Dajian, Xiao Jun, Guo Xiaoyu, Xu Shujuan, Niu Yuda, Jin Jingbo, Zhang Hui, Xu Xun, Li Legong, Wang Wen, Qian Qian, Ge Song, Chong Kang (2015). COLD1 Confers Chilling Tolerance in Rice. Cell.

[CR18] Nakhamchik A, Zhao ZY, Provart NJ, Shiu SH, Keatley SK, Cameron RK, Goring DR (2004). A comprehensive expression analysis of the Arabidopsis proline-rich extensin-like receptor kinase gene family using bioinformatic and experimental approaches. Plant Cell Physiol.

[CR19] Shiu SH, Bleecker AB (2001). Receptor-like kinases from Arabidopsis form a monophyletic gene family related to animal receptor kinases. P Natl Acad Sci USA.

[CR20] Silva NF, Goring DR (2002). The proline-rich, extensin-like receptor kinase-1 (PERK1) gene is rapidly induced by wounding. Plant Mol Biol.

[CR21] Vannini C, Locatelli F, Bracale M, Magnani E, Marsoni M, Osnato M, Mattana M, Baldoni E, Coraggio I (2004). Overexpression of the rice Osmyb4 gene increases chilling and freezing tolerance of Arabidopsis thaliana plants. Plant J.

[CR22] Wang QS, Sang XC, Ling YH, Zhao FM, Yang ZL, Li YF, He GH (2009). Genetic analysis and molecular mapping of a novel gene for zebra mutation in rice (Oryza sativa L.). J Genet Genomics.

[CR23] Xing YD, Guo S, Chen XL, Du D, Liu MM, Xiao YH, Zhang TQ, Zhu MD, Zhang YY, Sang XC, He GH, Wang N (2018). Nitrogen metabolism is affected in the nitrogen-DeficientRice mutant esl4 with a calcium-dependent protein KinaseGene mutation. Plant Cell Physiol.

[CR24] Yang TB, Ali GS, Yang LH, Du LQ, Reddy ASN, Poovaiah BW (2010). Calcium/calmodulin-regulated receptor-like kinase CRLK1 interacts with MEKK1 in plants. Plant Signal Behav.

[CR25] Yang TB, Chaudhuri S, Yang LH, Du LQ, Poovaiah BW (2010). A calcium/calmodulin-regulated member of the receptor-like kinase family confers cold tolerance in plants. J Biol Chem.

[CR26] Ye YY, Ding YF, Jiang Q, Wang FJ, Sun JW, Zhu C (2017). The role of receptor-like protein kinases (RLKs) in abiotic stress response in plants. Plant Cell Rep.

[CR27] Yoo SC, Cho SH, Sugimoto H, Li JJ, Kusumi K, Koh HJ, Iba K, Paek NC (2009). Rice Virescent3 and Stripe1 encoding the large and small subunits of ribonucleotide reductase are required for chloroplast biogenesis during early leaf development. Plant Physiol.

[CR28] Zhang T, Li YF, Ma L, Sang XC, Ling YH, Wang YT, Yu P, Zhuang H, Huang JY, Wang N, Zhao FM, Zhang CW, Yang ZL, Fang LK, He GH (2017). LATERAL FLORET 1 induced the three-florets spikelet in rice. P Natl Acad Sci USA.

[CR29] Zhou MQ, Shen C, Wu LH, Tang KX, Lin J (2011). CBF-dependent signaling pathway: a key responder to low temperature stress in plants. Crit Rev Biotechnol.

[CR30] Zhu XY, Guo S, Wang ZW, Du Q, Xing YD, Zhang TQ, Shen WQ, Sang XC, Ling YH, He GH (2016) Map-based cloning and functional analysis of YGL8, which controls leaf colour in rice (Oryza sativa). BMC Plant Biol 16:134 10.1186/s12870-016-0821-5 PMC4907030 27297403

