# Supplementary material for: Zebra leaf 15, a receptor-like protein kinase involved in moderate low temperature signaling pathway in rice
Source: Rice (N Y). 2019 Nov 15;12:83. doi: 10.1186/s12284-019-0339-1 (PMC6858429; doi:10.1186/s12284-019-0339-1)
Supplement: Supplementary file 1 — Additional file 1: Figure S1. The phenotype of wild type and z15 mutant. Figure S2. Transmission electron micrographs of chloroplasts at tiller stage. Figure S3. Agronomic traits of the wild type (‘Jinhui 10’) and z15 mutant at mature stage. Figure S4. Protein sequence alignment of Z15. Figure S5. Protein kinases domain profile. Figure S6. Transcriptome analysis and expression of genes involved in cold stress response. Figure S7. Sequencing method. [file 12284_2019_339_MOESM1_ESM.docx]

**Zebra leaf 15, a receptor-like protein kinase involved in moderate low temperature signaling pathway in rice**

Additional file 1

.


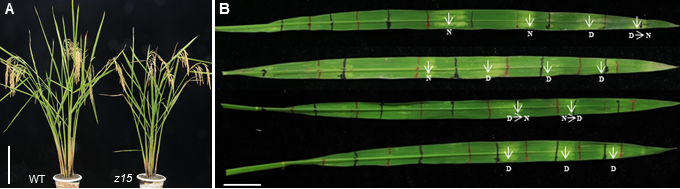


Figure S1 The phenotype of wild type and *z15* mutant.

A, The phenotype of wild type and *z15*at mature stage. Bar=30cm. B, The phenotype of *z15* leaf blade (The leaves were marked where the phenotype appears by red and black line in morning and evening respectively, (N: night; D: day)). Bar=1cm.


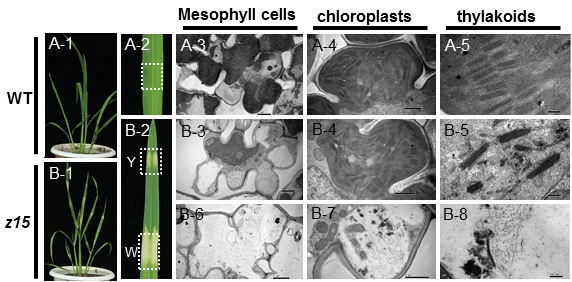


Figure S2 Transmission electron micrographs of chloroplasts at tiller stage.

A-1: The plant of WT; A-2: The leaf blade of WT; B-1: The plant of *z15*; B-2: The leaf blade of *z15*; Y: Yellow area of the *z15* leaf blade. W: White area of the *z15* leaf blade. A3, A-4, A-5: Transmission electron micrographs of chloroplasts of WT; B-3~B-8: Transmission electron micrographs of chloroplasts of *z15*.A-3,B-3,B-6:Bar=2μm; A-4,B-4,B-7:Bar=1μm; A-5,B-5,B-8:Bar=200nm.


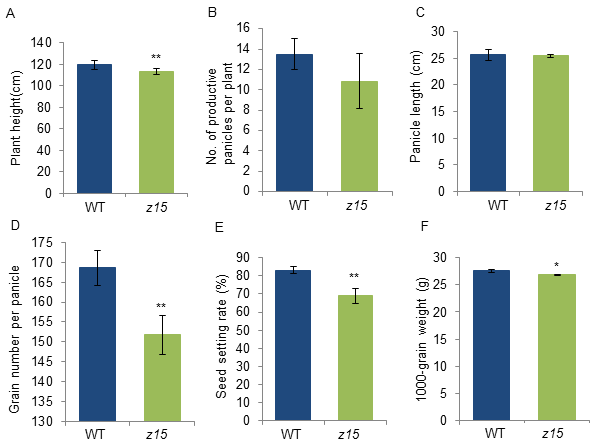


Figure S3 Agronomic traits of the wild type (‘Jinhui 10’) and *z15* mutant at mature stage.

A, Plant Height. B, No. of productive panicles per plant. C, Panicle length.D, Grain number per panicle. E, Seed setting rate. F,1000-grain weight. Values represent means±SD from 15-20biological samples. Asterisks indicate the significance of the differences between WT and *z15* as determined by Student’s t-test (*, 0.01≤ p <0.05; **, p < 0.01).


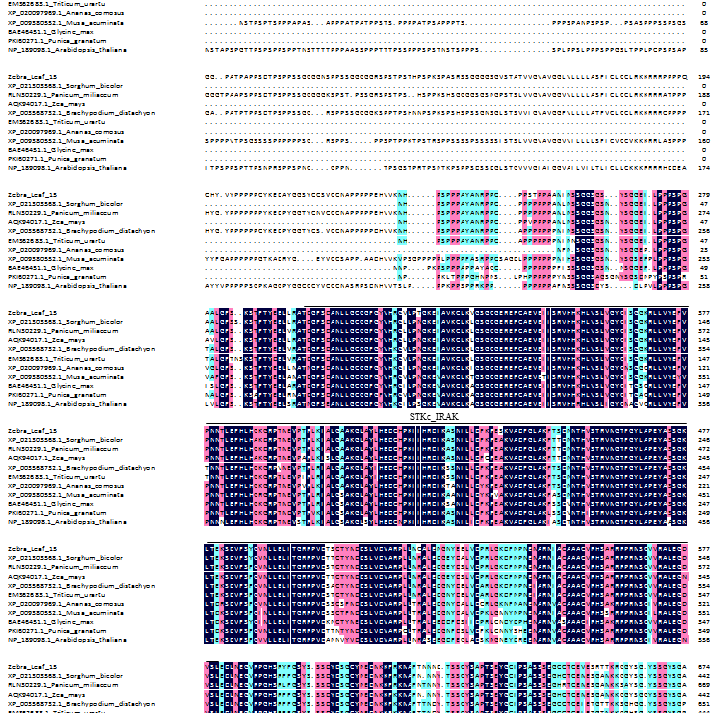


Figure S4 Protein sequence alignment of Z15.


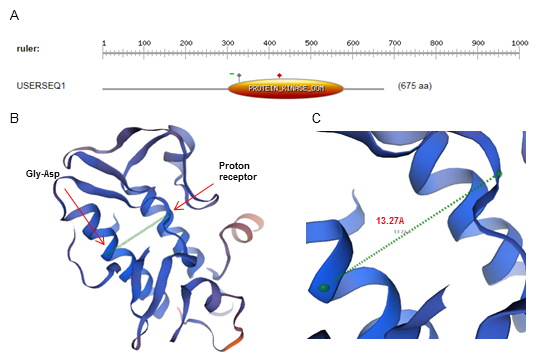


Figure S5 Protein kinases domain profile.

A, Putative conserved domains.B, The structure of Z15 protein. C, The Close-up of images from B.


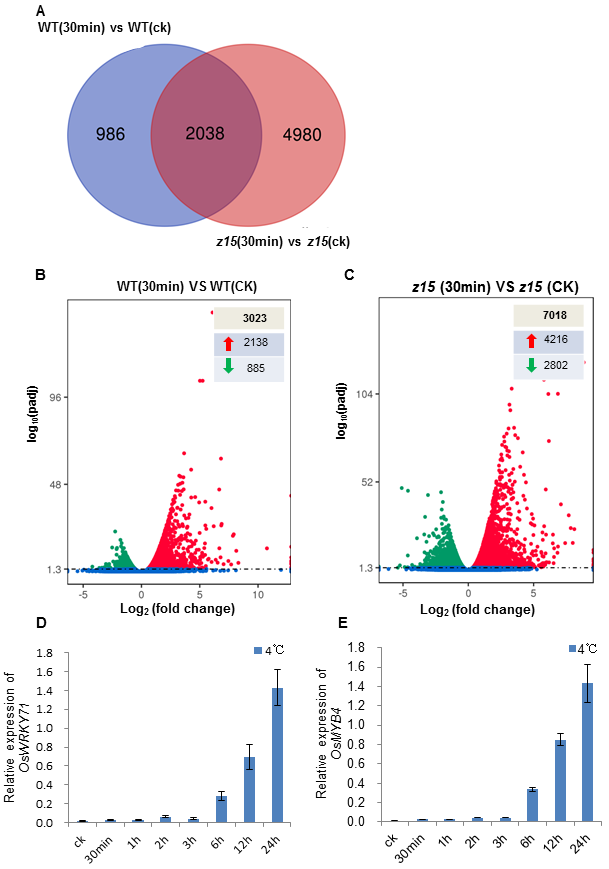


Figure S6 Transcriptome analysis and expression of genes involved in cold stress response.

A, Venn diagram showing common and specific DEGs numbers from different combinations displayed in the overlapping and non-overlapping regions, respectively. B, The Volcano plot of “WT (30min) vs WT (ck)”. C, The Volcano plot of “*z15* (30min) vs *z15* (ck)”. D, The expression of *OsWRKY71*at 4°C. E, The expression of *OsMYB4* at 4°C.


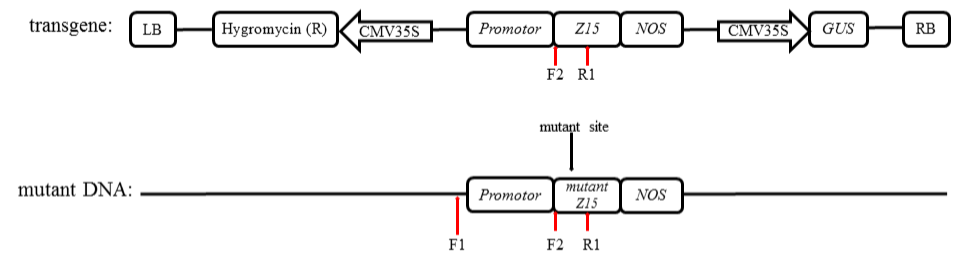


Figure S7 Sequencing method
