# Supplementary material for: Zebra leaf 15, a receptor-like protein kinase involved in moderate low temperature signaling pathway in rice
Source: Rice (N Y). 2019 Nov 15;12:83. doi: 10.1186/s12284-019-0339-1 (PMC6858429; doi:10.1186/s12284-019-0339-1)
Supplement: Supplementary file 2 — Additional file 2: Table S1. Primers used in the study. Table S2. DEGs annotated within the cold-response. Table S3. DEGs annotated within the cold-response. [file 12284_2019_339_MOESM2_ESM.docx]

Table S1. Primers used in the study

| Purpose | Primer name | Sequence | Remarks |
| --- | --- | --- | --- |
|  | nSSR516-F | GCGGATAGTCCGGATACGG |  |
| Mapping | nSSR516-R | GCTAGGTTGAAGGTCTAGAGC |  |
|  | Z15-13F | CTCTACCATCAGCCAGGCAG |  |
|  | Z15-13R | TGTGATGTACTGCTGATACTTAACAA |  |
| Complementary | Z15-COM-F | GCCggtaccCCTGGCTAGTTGCACCACAA | KpnΙ |
|  | Z15-COM-R | GCCctgcagGCAACTGCCAGAGCCAGTAGAT | PstΙ |
| Subcellular | Z15-S-F | GCCactagtATGTCGTCGCCGACCGCC | SpeΙ |
| localization | Z15-S-R | GCCggatccTGAGGCTCCGCTGTATCCTGAGC | BanHΙ |
| GUS expression | Z15-P-F | GCCggatccGATACCACGATAATTTATCTCCCATGT | BanHΙ |
|  | Z15-P-R | AGGccatggATCGCGCACCCCGCTCC | NcoΙ |
| In situ | Z15-ISH-F | GATCGTACAGCAGCTCTGACT |  |
|  | Z15-ISH-R | TAATACGACTCACTATAGGGGTGGAACACATCCATAACATAATAC |  |
|  | Action-F  Action-R  Z15Exp-F  Z15Exp-R | GACCCAGATCATGTTTGAGACCT  CAGTGTGGCTGACACCATCAC  CTTGGTTGACTGGGCAAGGC  CGGACAACCTGGCTCATGC |  |
| qRT-PCR | OsWRKY71-EXP-F | CCCTGCCCAAGAGCCTACTTCAG |  |
|  | OsWRKY71-EXP-R | CTGACGGTGCGGCACCACC |  |
|  | OsMYB4-EXP-F | GGTGACGGAGTCCTCGATGGC |  |
| *In vitro* enzyme activity assay | OsMYB4-EXP-R  Z15-Pet32a-F  Z15-Pet32a-R | GTCTTGCGCTTCGCCGGACTC  GACAGCCCAGATCTGGGTACCTGCTGCCTCCGGAAGAAGCG  TTGTCGACGGAGCTCGAATTCTGAGGCTCCGCTGTATCCTGAGC | KpnΙ  EcoRΙ |

Table S2. DEGs annotated within the cold-response.

| Gene  Name | Gene  Name | | log2Fold Change | | | Gene  Description |
| --- | --- | --- | --- | --- | --- | --- |
|  |  |  | WT  (30min VS ck) | *z15*  (30min VS ck) | |  |
| *OsNPR1*  *OsSAP1*  *OsTPP1*  *OsMAP1*  *OsCDPK14*  *OsWRKY71*  *OsMYB4* | | *LOC_Os01g09800*  *LOC_Os09g31200*  *LOC_Os02g44230*  *LOC_Os03g17700*  *LOC_Os11g07040*  *LOC_Os02g08440*  *LOC_Os04g43680* | 1.06  2.11  2.88 3.7462  2.079 1.3945  0.95693  3.36 2.46  2.42 | | BTBA1- Bric-a-BracTramtrack Broad Complex BTB domain with Ankyrin repeat region expressed  AN1-like zinc finger domain containing protein expressed  CPuORF22 - conserved peptide uORF-containing transcript expressed  Mitogen-Activated Protein  CAMK_CAMK_like.43-CAMK includes calcium Fcalmodulin depedent protein kinases expressed  *WRKY71* expressed  *MYB* family transcription factor putative expressed | |

Table S3. DEGs annotated within the cold-response.

| gene_name | gene_name | *z15*.CK | *z15*.30min | WT.CK | WT.30min |
| --- | --- | --- | --- | --- | --- |
| *OsNPR1*  *OsSAP1*  *OsTPP1*  *OsMAP1*  *OsCDPK14*  *OsWRKY71*  *OsMYB4* | *LOC_Os01g09800*  *LOC_Os09g31200*  *LOC_Os02g44230*  *LOC_Os03g17700*  *LOC_Os11g07040*  *LOC_Os02g08440*  *LOC_Os04g43680* | 4.88  25.42  0.25  17.00  21.68  16.91  4.18 | 5.95  137.89  3.00  40.31  29.66  84.30  6.49 | 3.86  31.81  0.50  11.36  15.79  12.92  3.05 | 8.35  144.16  3.49  50.17  32.14  156.95  16.90 |
